# Rapid Cartesian versus radial acquisition: comparison of two sequences for hepatobiliary phase MRI at 3 tesla in patients with impaired breath-hold capabilities

**DOI:** 10.1186/s12880-017-0203-y

**Published:** 2017-05-09

**Authors:** Johannes Budjan, Philipp Riffel, Melissa M. Ong, Stefan O. Schoenberg, Ulrike I. Attenberger, Daniel Hausmann

**Affiliations:** Department of Clinical Radiology and Nuclear Medicine, University Medical Center Mannheim, Medical Faculty Mannheim, Heidelberg University, Theodor-Kutzer-Ufer 1-3, 68167 Mannheim, Germany

**Keywords:** radial VIBE, Hepatobiliary MRI, Breathing artifact reduction, Liver-specific contrast agent

## Abstract

**Background:**

Hepatocyte-specific gadolinium based contrast agents (HSCA) provide substantial information for the classification of liver lesions in magnetic resonance imaging (MRI). However, breathing artifacts which reduce image quality and diagnostic confidence of hepatobiliary phase acquisitions are regularly observed in clinical routine. The aim of this study was to evaluate two approaches to reduce breathing artifacts for hepatobiliary phase imaging.

**Methods:**

Twenty minutes after administration of a HSCA (gadoxetic acid), a T1-weighted VIBE sequence with radial k-space sampling (radialVIBE, 180 s acquisition time in free breathing) and a highly accelerated Cartesian VIBE with Dixon fat separation (CD-VIBE, CAIPIRINHA acceleration with *r* = 2 × 2, breath-hold 8–10 s) were acquired in 35 patients (12 female, 57 ± 13 years), who showed breath-holding difficulties in early phases of the examinations. Image quality (image sharpness, noise, artifacts, homogeneity of fat saturation, bile duct delineation and overall image quality) as well as conspicuity and liver-to-lesion signal intensity (SI) ratios of focal liver lesions were assessed for both radial- and CD-VIBE.

**Results:**

Overall image quality was rated good to excellent for both sequences, while CD-VIBE was preferred in most cases. Though radialVIBE received better results regarding image noise and artifacts, both sequences were rated equally regarding bile duct delineation and sharpness. Focal liver lesion (*n* = 42) conspicuity was rated significantly better and SI-ratios were significantly higher on CD-VIBE (2.45 ± 1.44 vs. 1.61 ± 0.70 in radialVIBE, *p* = 0.0001). In three patients, CD-VIBE was rated non-diagnostic due to severe breathing artifacts, while radialVIBE was diagnostic in those patients.

**Conclusion:**

Both highly accelerated Cartesian as well as radial acquisition techniques provide good to excellent image quality in hepatobiliary phase MRI. In comparison, CD-VIBE offered better overall image quality and liver lesion conspicuity. However, radialVIBE was a valuable alternative in patients unable to sustain even short breath-hold intervals. Further studies including lager patient cohorts are desirable to allow a transfer of these results to a general patient population.

## Background

Magnetic resonance imaging (MRI) plays a major role in the diagnostic workup of focal liver lesions, both for detection as well as differentiation. Besides functional techniques like diffusion weighted imaging (DWI), hepatocyte-specific gadolinium based contrast agents (HSCA) can provide substantial information for the detection and classification of focal liver lesions, as HSCA allow the evaluation of hepatocyte metabolism [1]. Detection of small lesions is crucial to assess the disease burden in patients suffering from hepatocellular carcinoma (HCC) [2], particularly when imaging is used for selection of liver transplant candidates. A multicenter study in which HSCA-enhanced liver MRI was compared to contrast-enhanced computed tomography (CT) found MRI to be superior in the detection of lesions smaller than 1 cm [3].

Unfortunately, breathing artifacts which may impair image quality and thus decrease diagnostic confidence are regularly observed in clinical routine, with small lesions likely to be missed. Breathing artifacts due to patients’ limited breath-holding capabilities are a main cause for reduced diagnostic quality, which is reported to occur in up to 20% of patients when using breath-hold intervals of 23 s [4]. Previous studies on dynamic arterial phase imaging found that patients receiving HSCA particularly suffered from breath-holding difficulties, probably due to their typically reduced overall clinical condition [5]. Sufficient hepatocellular HSCA uptake is usually seen several minutes after injection. Thus, hepatobiliary phases are typically acquired not earlier than 10 min p.i., and an interval of 20 min p.i. is widely used in clinical routine [2, 6, 7]. Especially critically ill patients might get exhausted after such relatively long in-scanner times, worsening their already reduced breath-holding capabilities.

In this context, sequences with radial read-out seem to be appealing, as the influence of repetitive motion (i.e. breathing) on image quality is reduced compared to Cartesian approaches [8]. Radial k-space sampling leads to higher sampling density of the central k-space parts and to undersampling of k-space periphery, reducing the influence of motion-induced phase errors on image quality. On the other hand, new parallel imaging techniques allow highly accelerated acquisition, resulting in substantially shorter breath-hold intervals [9, 10].

The aim of this study was to evaluate and compare these different approaches for the reduction of motion artifacts in the hepatobiliary phase–a T1-weighted (T1w), volumetric-imaging-breath-hold-examination-(VIBE) sequence with radial k-space sampling (radialVIBE) acquired in free breathing and a highly accelerated Cartesian VIBE with Dixon fat separation (CD-VIBE) acquired in a short breath-hold.

## Methods

### Patients

**Fig. 1:**
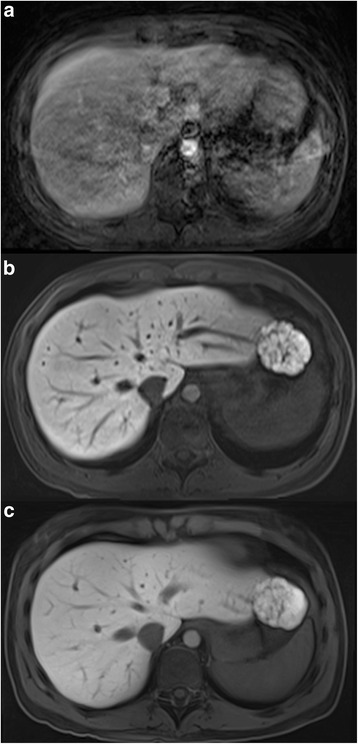
Example of a patient showing severe breathing artifacts in the dynamic arterial phase sequence (**a**) who received both CD-VIBE (**b**) and radialVIBE (**c**) sequence in the hepatobiliary phase. Both hepatobiliary phase sequences received excellent ratings regarding image quality

The institutional review board (Medizinische Ethikkommmision 2, Medizinische Fakultät Mannheim) waived the requirement of informed patient consent for this retrospective study. Using a database search, patients who underwent a HSCA liver exam on a specific MR scanner system were identified. The exams were screened for breathing artifacts in the early examination steps (pre-contrast breath-hold sequences as well as the dynamic arterial sequence). A total of 35 patients (12 female, 57 ± 13 years) who showed breathing artifacts in the early phases were included (Fig. 1). Indication for the liver exams were suspected hepatocellular carcinoma, liver metastasis or further differentiation of focal liver lesions that were detected by sonography or computed tomography.

### MR imaging

The examinations were acquired on a single 3 T MR scanner (MAGNETOM Skyra, Siemens Healthineers, Germany) using an 18-element body matrix coil and an inbuilt 32-element spine matrix coil. The liver examination protocol included T2w and DWI as well as native and dynamic T1w sequences after the injection of the HSCA (0.025 mmol/kg KG Primovist, Bayer HealthCare, Germany). The HSCA was injected manually at a rate of 1.5 mL/s, followed by a 20 mL saline flush. Hepatobiliary phase imaging was performed 20 min after injection of the HSCA. A highly-accelerated sequence with Dixon fat separation (CAIPIRINHA-Dixon-VIBE, CD-VIBE) and a VIBE sequence with radial k-space read-out (radialVIBE) and spectral fat suppression were acquired. While the breath-hold duration for CD-VIBE was 8–10 s, radialVIBE was acquired during free breathing. In most cases (*n* = 25), CD-VIBE was acquired directly prior radialVIBE. Sequence parameter details are shown in Table 1.

**Table 1:** Overview over the sequence parameter details

	CD-VIBE	radialVIBE
*TE/TR [ms]*	1.7/4.2	2.5/5.5
*Flip angle*	30°	12°
*Fat suppression technique*	Dixon	Spectral
*In-plane resolution [mm* ^*2*^ *]*	1.2 × 1.2	1.2 × 1.2
*Slice thickness [mm]*	3	3
*PAT*	2 × 2 (CAIPIRINHA)	-
*Radial views*	-	14,000
*Acquisition time*	8–10 s in single breath-hold	180 s during free breathing

*PAT* parallel acquisition technique

### Image analysis

Two radiologists with 7 and 4 years of experience in abdominal MRI assessed image sharpness, noise, artifacts, homogeneity of fat saturation, bile duct delineation as well as the overall image quality of both CD-VIBE and radialVIBE on a 5-point Likert-scale in consensus, 1 indicating worst, 5 the best score in respect to image quality (e.g. for overall quality: 5, excellent; 4, good image quality; 3, moderate image quality with slightly detrimental artifacts; 2, poor image quality with artifacts yet still diagnostic; and 1, non-diagnostic image quality; and for image artifacts: 5, no image artifacts; 4, minimal image artifacts; 3, moderate image artifacts; 2, from moderate to severe image artifacts; 1, severe image artifacts). Even though the order of sequences was randomized in the image quality reading, the readers could in fact not be blinded to the respective sequence technique due to inherent differences in their appearances (e.g. streak artifacts indicating radialVIBE).

If focal liver lesions were visible on the hepatobiliary phase images, lesion conspicuity was assessed for a maximum of three lesions per patient on a 5-point Likert-scale. If more than three lesions were detectable, the largest as well as the two smallest lesions were assessed. CD-VIBE and radialVIBE were evaluated separately, lesions were marked on both sequences and a final side-by-side comparison was performed to ensure that identical lesions were selected. In the side-by-side comparison, both readers also chose their overall preferred sequence. For quantitative analysis, average SI was measured in an oval region-of-interest (ROI) in the lesion as well as the adjacent liver parenchyma. ROIs were placed on the slice on which the lesion showed the largest dimensions. For lesions with reduced HBCA uptake, signal ratio was calculated as SI liver parenchyma/SI lesion, for hyperintense lesions SI lesion/SI liver parenchyma. Largest diameter was measured for all included lesions on both sequences.

### Statistical analysis

The statistical analysis was performed using JMP 11.0 (SAS Institute, Cary, NC). For the comparison of image quality scores, intensity ratios and lesion diameters, two-tailed paired Wilcoxon signed rank tests were applied. Image quality parameters as well as SI ratios are given as mean values. Lesion diameters are given as median values. Two-tailed *p*-values of < 0.05 were considered statistically significant.

## Results

**Fig. 2:**
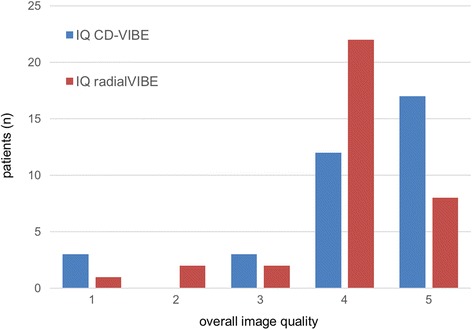
Overview of overall image quality ratings for both CD-VIBE (*blue*) and radialVIBE (*red*)

In the evaluation of the different image quality parameters, both sequences received good to excellent ratings: Regarding image sharpness (CD-VIBE 4.3, radialVIBE 4.2; *p* = 0.49) and delineation of bile ducts (CD-VIBE 4.4, radialVIBE 4.2, *p* = 0.19), no statistically significant differences were found between both sequences. Regarding image noise (CD-VIBE 4.0, radialVIBE 4.6, *p* = 0.0002) and image artifacts (CD-VIBE 3.7, radialVIBE 4.1, *p* = 0.02), radialVIBE received statistically significantly higher ratings. Fat saturation homogeneity was rated superior for CD-VIBE compared to radialVIBE (4.3 vs. 3.7, *p* = 0.0002). For overall image quality, 17/35 of the CD-VIBE acquisitions were rated with the highest rating of 5, whereas only 8/35 examinations received a rating of five for radialVIBE (Fig. 2). However, overall image quality was equal for both sequences with an average overall image quality of 4.1 for CD-VIBE and 3.9 for radialVIBE, which was not statistically significant different in the pair-wise analysis (*p* = 0.18). Both readers preferred CD-VIBE in the side-by-side comparison in most cases (24/35 for reader 1, 25/35 for reader 2).

**Fig. 3:**
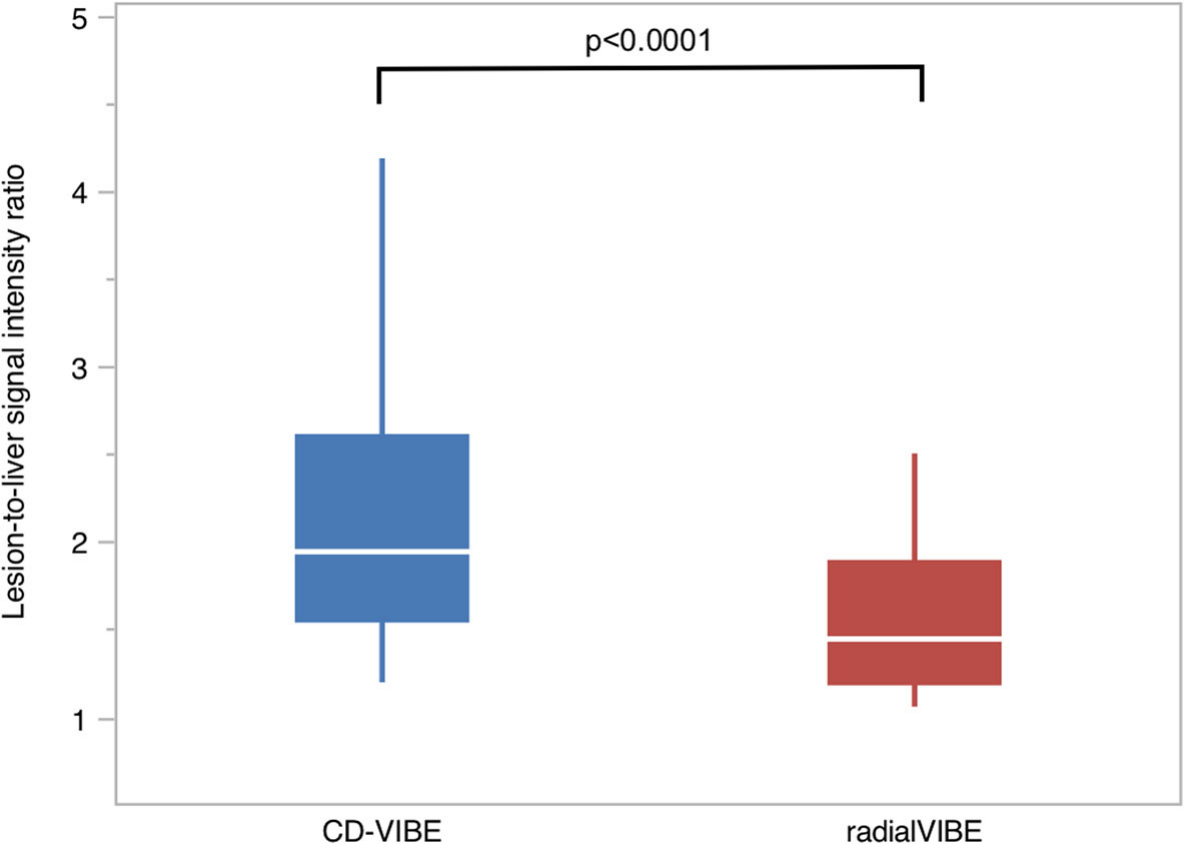
Box-plot depicting liver-to-lesion contrast for both sequences with CD-VIBE showing statistically significantly higher ratios in the pairwise comparison

A total of 42 liver lesions were found in 23 patients with a median lesion size of 16 mm (range 5-124 mm) as measured in the CD-VIBE sequence. Regarding lesion conspicuity, CD-VIBE received statistically significantly higher ratings (4.6 vs. 3.8 for radialVIBE, *p* < 0.0001). Similar results were obtained in the quantitative analysis of SI ratios, in which CD-VIBE showed an average SI ratio of 2.45 ± 1.44, while the SI ratio in radialVIBE was statistically significantly lower with 1.61 ± 0.70 (*p* < 0.0001, Fig. 3), indicating a lower contrast between lesion and surrounding liver parenchyma for radialVIBE. Regarding largest lesion diameter, no statistically significant differences between the sequences was found (median lesion size in radialVIBE 17 mm, *p* = 0.93).

The overall image quality was rated non-diagnostic (0) for CD-VIBE in three patients. In those patients, the equivalent radialVIBE sequences received diagnostic ratings (with 2, 3 and 3, respectively).

## Discussion

**Fig. 4:**
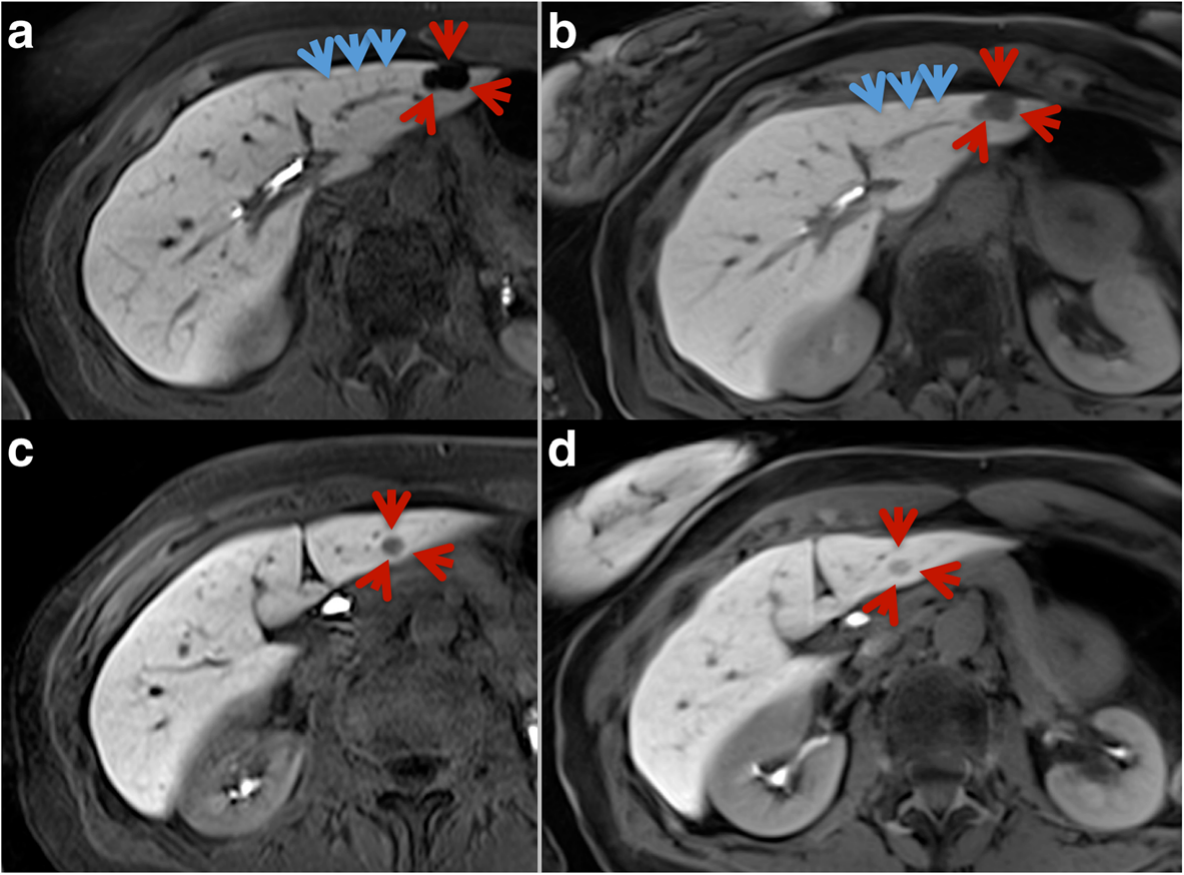
In the direct comparison of CD-VIBE (**a**, **c**) and radialVIBE (**b**, **d**), CD-VIBE shows higher intraparenchymal detail (*blue arrows*) as well as better visual lesion delineation (*red arrows*)

**Fig. 5:**
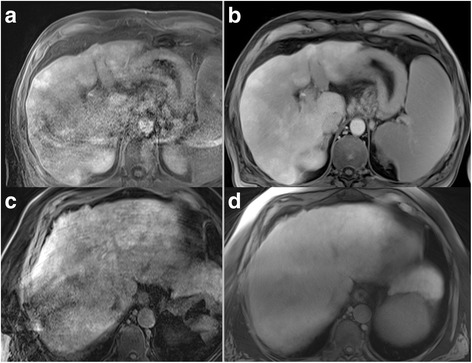
Examples of two patients with non-diagnostic image quality in CD-VIBE (**a**, **c**) due to breathing artifacts. In those patients, radialVIBE (**b**, **d**) achieved diagnostic image quality (rated as 3, top and 2, bottom on a 5-point Likert scale)

**Fig. 6:**
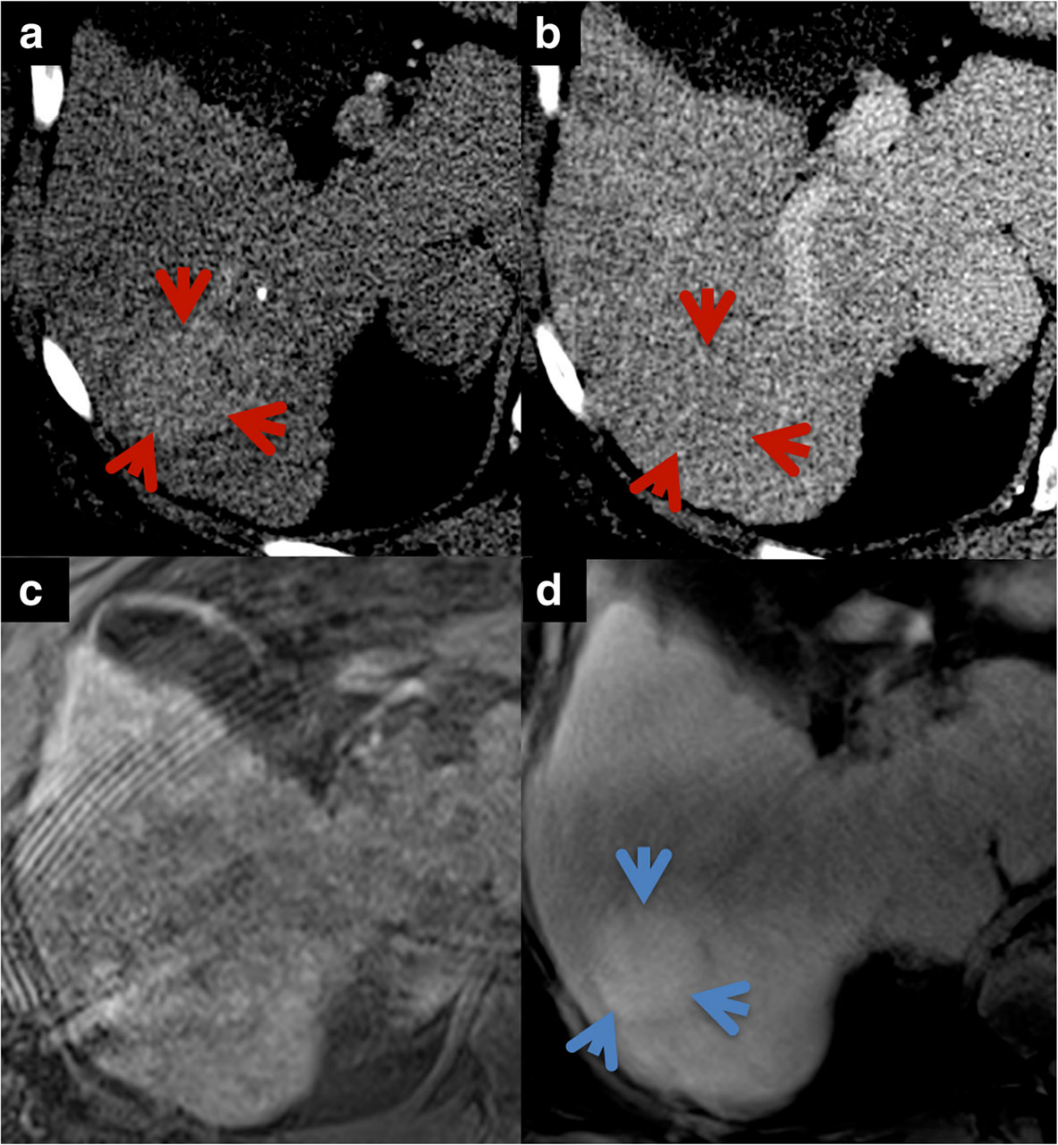
Example of patient presenting with early enhancing (**a**, *red arrows*) hepatic lesion with rapid contrast wash-out in the portal-venous phase on CT images (**b**). Additional hepatobiliary phase HSCA-enhanced MRI was acquired to assess lesion uptake. CD-VIBE (**c**) was non-diagnostic due to motion artifacts in this patient with impaired breath-holding capability. Despite of clearly visible increased lesion uptake on radialVIBE (**d**, *blue arrow*), low-grade HCC was confirmed histopathologically following CT-guided biopsy and was subsequently treated by TACE with curative intention

The majority of patients with breath-holding difficulties in the early examination phases were able to maintain the substantially shorter breath-hold in the accelerated Cartesian sequence used in the hepatobiliary phase. The superior visual lesion delineation and the higher intraparenchymal detail were main reasons why CD-VIBE was the preferred sequence in a side-by-side comparison in most cases (Fig. 4). However, three patients showed breath-holding difficulties despite the short breath-hold interval, resulting in non-diagnostic hepatobiliary phase images using CD-VIBE. RadialVIBE was able to generate diagnostic images in those patients (Figs. 5 and 6).

Most studies that previously assessed the image quality of robust radial sequences in comparison to standard techniques were conducted on early arterial phase sequences. Those studies revealed a superiority of radial approaches in patients with reduced breath-holding capabilities [11].

To our knowledge, there are just two studies to compare radial and standard Cartesian sequences in the late hepatobiliary phase, which is crucial for lesion detection, particularly in suspected HCC and metastasis. A study on hepatobiliary phase MRI in patients with reduced breath-holding capabilities compared image quality and diagnostic performance of a free-breathing radial 3D-gradient-echo (GRE) sequence and a Cartesian breath-hold 3D-GRE sequence and found that liver surface sharpness was improved using the radial acquisition approach. Additionally, overall image quality of the radial acquisition was superior, while bile duct visualization did not differ significantly between both sequences. Lesion detection was similar for both techniques [8]. However, the acquisition time of the Cartesian sequence in this study was 24 s. As stated earlier, breath-hold intervals of >20 s are challenging for patients with reduced breath-holding capabilities. In our study, the acquisition time of CD-VIBE was reduced substantially to as short as 10 s with only few patients struggling to hold their breath during this short interval.

Another study to compare a fat-saturated 3D T1w GRE sequence using a breath-hold technique (cGRE) with a flip angle of 12° to a fat-saturated T1w spin-echo sequence using a radial read-out during free breathing (rSE) for hepatobiliary phase HSCA-enhanced MRI revealed that rSE images were equal to or better than cGRE images in patients who struggled to hold their breath. The study found higher relative liver signal intensity in the rSE sequence [12]. The significantly higher lesion to liver SI ratios of CD-VIBE compared to radialVIBE measured in our study are most likely attributable to the increased flip angle used in the CD-VIBE (30° in CD-VIBE versus 12° in radialVBE). The Dixon fat separation approach used in the CD-VIBE allowed the employment of higher flip angles within normal mode whole body specific absorption rate (SAR) ranges (i.e. < 2 W/kg bodyweight). In contrast, using higher flip angles in radial acquisition to increase liver-to-lesion contrast was limited due to SAR limitations and acquisition time constraints.

Additionally, our study found that fat-saturation of radialVIBE was inferior to that of CD-VIBE. This is likely due to the spectral approach used in radialVIBE in comparison to the Dixon fat separation technique used in CD-VIBE. The Dixon fat separation approach was shown to provide improved fat suppression in previous studies carried out on the same MR system [13]. However, inhomogeneous fat suppression in the radialVIBE sequence usually occurred in regions outside the liver and was most pronounced in subcutaneous fat, thus mostly not affecting diagnostic image quality. Regarding visual image noise and artifacts, the radialVIBE sequence received better ratings, however, both parameters were rated good for CD-VIBE as well. In the radialVIBE sequence, typical streak artifacts were mostly seen in the center of the images. While they did not impair image quality in the liver, such artifacts may affect assessment of adjacent organs like portal vein and confluence or the pancreatic head. Finally, we revealed good to excellent conspicuity of bile ducts for both sequences with slight advantages for CD-VIBE, which may be important during late hepatobiliary phase to exclude biliary anastomotic leakage following liver transplantation [7].

Our study has a number of limitations. First and probably of most importance, the study population was relatively small for a diagnostic study, thus potentially hindering transfer of our results to a general patient population. Thus, larger studies including larger patient cohorts are required to confirm our results. Second, the patient group and underlying liver disease were heterogeneous. Studies that specifically assess different tumor entities would be useful to evaluate the performance of both techniques in lesions with increased and decreased uptake compared to healthy liver tissue, respectively. In this context, evaluation of uptake of regenerative nodules would be interesting being particularly useful for early identification of malignant transformation into low-grade HCC. Another potential limitation is the above mentioned flip angle difference between both sequences. An improved radial sequence to employ higher flip angles and thus increase in lesion contrast would be desirable.

## Conclusion

We may conclude that both highly accelerated Cartesian as well as radial acquisition techniques provide good to excellent image quality in hepatobiliary phase MRI. In comparison, CD-VIBE offers better overall image quality and focal liver lesion conspicuity, which made it the preferred sequence in a direct side-by-side comparison. However, radialVIBE is a valuable alternative in patients unable to sustain even short breath-hold intervals.

## Abbreviations

CT: Computed tomography
GRE: Gradient-echo
HCC: Hepatocellular carcinoma
HSCA: Hepatocyte-specific gadolinium based contrast agents
MRI: Magnetic resonance imaging
ROI: Region-of-interest
SAR: Specific absorption rate
SI: Signal intensity
T1w: T1-weighted
TACE: Transarterial chemoembolization
